# A double-blind intervention trial in healthy women demonstrates the beneficial impact on *Bifidobacterium* with low dosages of prebiotic galacto-oligosaccharides

**DOI:** 10.3389/fnut.2024.1440319

**Published:** 2024-08-19

**Authors:** Ellen Looijesteijn, Marieke H. Schoemaker, Maartje van den Belt, Eric R. Hester, Guus A. M. Kortman, Mirre Viskaal-van Dongen, Arjen Nauta

**Affiliations:** ^1^FrieslandCampina, Amersfoort, Netherlands; ^2^Wageningen Food and Biobased Research, Wageningen University and Research, Wageningen, Netherlands; ^3^NIZO food research B.V., Ede, Netherlands

**Keywords:** galacto-oligosaccharides, GOS, *Bifidobacterium*, gut microbiota, prebiotic, supplement, women

## Abstract

**Introduction:**

Galacto-oligosaccharides (GOS) are well-substantiated prebiotic substrates. Multiple studies have demonstrated a positive impact of GOS on gut microbiota composition and activity, so-far mainly related to *Bifidobacterium*. However, data on the beneficial impact at lower dosages in a healthy female population are limited. The primary aim of the current study was to reveal the effect of low dosages (1.3 and 2.0 g) of GOS on fecal *Bifidobacterium* abundance in healthy women. Other outcomes included the effect of low dosage of GOS on overall fecal microbiota composition and on self-perceived GI comfort, sleep quality and mental wellbeing.

**Method:**

Eighty-eight healthy women (42–70 years, BMI 18.7–30 kg/m^2^) were included in this randomized, parallel, double-blind study of 6 weeks. The participants were stratified for fiber intake, BMI and age and randomized to consume either 1.3 or 2.0 g of GOS per day for 3 weeks after a control period of 3 weeks without any intervention. Fecal samples were collected for shotgun metagenomics sequencing at the start (t = −3) and end (t = 0) of the control period and at the end of the intervention period (t = 3). Self-perceived gut comfort, sleep quality, and mental wellbeing were assessed weekly. Hierarchical clustering of principal components was applied to data collected from study participants.

**Results:**

The relative abundance of *Bifidobacterium* in feces increased significantly after 3 weeks of daily consumption of both 1.3 g (*p* < 0.01) and 2.0 g GOS (*p* < 0.01). This was accompanied by a significant shift in the overall microbiota composition for the dosage of 2.0 g GOS (*p* < 0.01). Participants that showed a larger increase in *Bifidobacterium* in the intervention period compared to the change in *Bifidobacterium* in the control period, defined as responders, showed a significant overall difference in initial fecal microbiota composition as compared to non-responders (*p* = 0.04) and a trend towards lower baseline levels of *Bifidobacterium* in responders (*p* = 0.10).

**Conclusion:**

Daily consumption of a low dose of GOS can lead to an increase in the relative abundance of *Bifidobacterium* in feces of healthy women. Additionally, with 2.0 g GOS, the enrichment of *Bifidobacterium* is accompanied with a shift in the overall microbiota composition.

**Clinical trial registration**: clinicaltrials.gov, identifier NCT05762965.

## 1 Introduction

A resilient gut microbiota is important for gut comfort and maintaining overall human health. Disturbances in gut microbiota are associated with several medical conditions, including intestinal diseases/infections and metabolic diseases (1, 2). Ageing is also associated with changes in the gut microbiota, such as a reduction of beneficial members of the genus *Bifidobacterium* (3). The age-related changes in the gut microbiota are associated with the health status of ageing adults (3–6). Emerging evidence indicates that the gut microbiota not only has a local impact but also a systemic impact through gut-organ axes such as the gut-brain axis (7, 8). Via the gut-brain axis, gut microbiota, including *Bifidobacterium*, might influence brain related functions such as mental wellbeing and sleep (9, 10).

Prebiotics are defined as substrates that are selectively utilized by host microorganisms conferring a health benefit (11). Acting as substrates, prebiotics can beneficially impact gut microbiota composition and activity, i.e., by enriching beneficial microorganisms (12). Galacto-oligosaccharides (GOS) are among the most established prebiotics (11). Many studies have shown that GOS impact gut microbiota in various target groups (13–23) and exert a beneficial role on gut microbiota composition and activity, and health outcomes (17, 18, 20). Primary outcomes varied from immune read-outs (22, 24, 25), iron absorption (15), mental wellbeing (17) and gut health (20, 26, 27). An increase of *Bifidobacterium* abundance is robustly reported in all these studies. Bifidobacteria have been linked to a healthy, resilient gut microbiota (12, 16, 28, 29) and are considered as beneficial bacteria in the gut because of the production of health promoting metabolites, such as short chain fatty acids (28, 30). Depletion of *Bifidobacterium* has been linked to several diseases, including inflammatory bowel diseases and irritable bowel syndrome (31–33).

Healthy females, aged 40 and above, are frequent users of supplements to support gut and digestive health (34). As supplements in the form of capsules or tablets can only accommodate a limited amount of ingredients, there is an interest to substantiate low dosages of GOS. So far, only a limited number of studies addressed the effectiveness of low daily dosages (≤2 g) of GOS on gut microbiota composition in a healthy population. Tamai et al., (1992) described the bifidogenic effect of 20 days consumption of 2 g GOS per day in healthy males (25–60 years) (35). Also 1-g GOS per day for 21 days increased levels of bifidobacteria in a study with healthy men (26–57 years) (36). In another study, daily consumption of 1.72 g GOS significantly increased fecal levels of *Bifidobacterium* in a study population of healthy adults (18–60 years) (13). These small-scale studies applied cultivation-dependent methods or only focused on specific microbiota members, which limits these studies’ ability to investigate the broader impact of low dosages of GOS on the microbiome. Therefore, the primary objective of this study was to determine the effect of a 3-week intervention, with either 1.3 or 2.0 g of prebiotic GOS, on the relative abundance of *Bifidobacterium* in feces of healthy female adults between 40 and 70 years. Secondly, we aimed to investigate the effect of both dosages of GOS on overall fecal microbiota composition. Tertiary outcomes of the study were the effects of low dosages of GOS on self-perceived GI comfort, sleep quality and mental wellbeing.

## 2 Materials and methods

### 2.1 Study design and study participants

The study was designed as a double blind, randomized trial with two parallel intervention groups. After a control period of 3 weeks without intervention, participants consumed the intervention product for 3 weeks. One group received 1.3 g of GOS per day and the other 2.0 g of GOS per day during the intervention period. The impact of GOS supplements on study outcomes was assessed using a within-subject design, comparing the intervention period with the control period. This design was chosen because of the large inter-individual variation in gut microbiota composition. During the study, fecal samples were collected to determine the abundance of *Bifidobacterium* and the gut microbiota composition (primary and secondary outcome, respectively) and questionnaires were completed on sleep, mental-wellbeing, and gastro-intestinal (GI) comfort (tertiary outcome).

Ethical approval for the study protocol was obtained from the Medical Ethical Committee of Oost-Nederland on 2 March 2023. The study was registered at clinicaltrials.gov (identifier NCT05762965). The trial was conducted at Wageningen Food and Biobased Research (WFBR), the Netherlands, between May and June 2023. The study was performed in accordance with the principles of the Declaration of Helsinki and that of the Medical Research Involving Human Subjects Act. Written informed consent was obtained from all participants prior to inclusion.

### 2.2 Study participants

Participants were recruited in the area of Wageningen, the Netherlands between mid-March and mid-May 2023. Participants were eligible for inclusion when they were healthy women between 40 and 70 years, with a BMI between 18.5 and 30 kg/m^2^. Exclusion criteria for the study were having a gastro-intestinal disease, a history of intestinal surgery, diabetes mellitus, use of medication that could potentially influence the study results, constipation, cow’s milk allergy, lactose intolerance, slimming, and weight loss or weight gain of more than 5 kg in the month prior to screening, pregnancy and lactation, use of drugs and intake of alcoholic beverages of more than 7 glasses a week. Participants were also excluded when using pre-, pro- and/or synbiotics within 4 weeks before the start of the study, when using antibiotics less than 3 months before the start of the study, and when participating in another clinical trial at the same time. Prior to the study, information meetings were organized to explain the background, objectives, and study procedures to participants. After participants signed the informed consent, weight and height were measured to determine their BMI. Furthermore, the participants filled in a screening questionnaire about their health, lifestyle, and fiber intake. This questionnaire also included questions about specific diets, such as veganism, and about the intake of nutritional supplements.

Eighty-eight women were enrolled in the study. These participants were stratified in groups based on fiber intake at screening, BMI, and age. Within the 8 different strata, participants were randomly allocated by the study coordinator of WFBR to one of the two intervention arms (1.3 and 2.0 g of GOS), using a random number generator.

### 2.3 Sample size calculation

For the sample size calculations ANOVA with repeated measures, within factors, was used in G*Power (version 3.1.9.7), using a power of 0.8, a significance level alpha of 0.05, 3 repeated measures (the 3 time points), an effect size of 0.3, and an assumed standard deviation (SD) of 1. A factor 0 was chosen for the correlation among repeated measures as a worst-case scenario. With these data, sample size calculation indicated the need for about 38 participants per treatment group. Anticipating a drop-out rate of about 15%, 44 participants were included per treatment group. This resulted in 88 participants.

### 2.4 Intervention product

Biotis® GOS (FrieslandCampina Ingredients, Amersfoort, Netherlands) was used as intervention product in the trial. This product is a powder containing a mixture of galacto-oligosaccharides. For the two different treatment groups, the GOS powder was packaged in small sachets containing either 1.3 g of GOS (1.5 g powder) or 2.0 g of GOS (2.2 g powder). All sachets were uniformly packaged, with a coded label that corresponded to one of the two arms of the study. Both participants and researchers were fully blinded to the treatments. During the intervention period, study participants took their intervention product once a day, during breakfast. Participants returned any unused study products to the study site. The number of returned sachets was used to determine study compliance (number of sachets taken during the study/number that should have been taken).

### 2.5 Microbiota analysis

The participants collected one fecal sample at the beginning of the control period (t = −3 weeks), one sample at the end of the control period (t = 0 weeks) and one sample at the end of the intervention period (t = 3 weeks). After collection, the fecal sample was immediately frozen in the home freezer of the participants. After collection of the final sample, stool samples were transported frozen to the study site and stored at −80°C until further analysis.

DNA isolation, library preparation, and sequencing were performed by BaseClear, Leiden, Netherlands. Fecal samples were processed using the ZymoBIOMICS™ 96 MagBead DNA Kit (D4302, Zymo Research) according to the manufacturer’s instructions with minor modifications to extract DNA. DNA samples were subjected to Illumina Nextera XT library preparation. The sequencing libraries obtained were sequenced on a NovaSeq 6000 instrument with paired-end 150 nt sequencing protocol. FASTQ read sequence files were generated using bcl2fastq2 version 2.18, which includes Illumina Chastity quality filtering with default settings. Subsequently, reads containing adapters and/or PhiX control signal were removed using an in-house filtering protocol. The second quality assessment was based on the remaining reads using the FASTQC quality control tool version 0.11.8.

At NIZO food research B.V. (Ede, Netherlands), read quality was checked using FastQC and reports generated using MultiQC. The number of sequencing reads per sample were analyzed for sufficient sequencing depth and to identify any samples significantly deviating from the average read depth (>3 SD below the mean was considered significant). After checking the samples for quality, sequencing reads were classified with a pipeline based on the Kraken2 taxonomic classifier software using the standard Kraken2 database (accessed March 2023) (37). Species abundances were adjusted using the Bracken software (38). Default settings were used apart from the Kraken2 confidence parameter which was set to 0.9 to reduce the number of false positives. A species abundance table from the output was prepared for downstream analysis. Finally, a principal coordinate analysis (PCoA), utilizing a Bray–Curtis dissimilarity matrix on all samples, was performed to visually inspect for significant deviations in the microbiota composition.

### 2.6 Questionnaires

GI comfort, stool frequency, sleep quality and mental wellbeing were determined weekly throughout the study using online questionnaires. Those online questionnaires also included monitoring of supplement compliance and adverse events.

The self-reported questionnaire to assess GI comfort consisted of six questions evaluating stool frequency, bloating, abdominal pain, flatulence, constipation, and diarrhea in the previous 7 days. A 4-point Likert scale (0 = none, 1 = present but tolerated, 2 = present interfering but not preventing activities, 3 = preventing daily activities) was used to score the different items, as has been applied before in adults with self-reported complaints (19, 24). The total intestinal comfort score (range 0–15) was calculated by summing up the scores of all items except for stool frequency; this total score was used in the analyses. Stool frequency was analyzed separately, and scored on a scale of 0–3, where a score of 0 represented “5 or more stools per week,” a score of 1 represented “3–4 stools per week” a score of 2 represented “2 stools per week,” and a score of 3 represented “one or less stools per week.” Sleep quality was assessed using the Athens Insomnia Scale (AIS). This is a self-reported, psychometric, validated questionnaire designed to quantify sleep difficulty based on the ICD-10 criteria (39). Each of the eight items of the questionnaire were scored on a scale of 0–3, with 0 corresponding to “no problems at all” and 3 corresponding to “very serious problem.” The total sum of scores (range 0–24) was used in the analyses. Mental wellbeing was determined using the self-reported DASS-21 questionnaire which is derived from the DASS-42 (40), and is designed and validated to measure depression, anxiety, and stress in the previous 7 days. The applied validated Dutch version included 7 questions for each of the three emotional states scored on a 4-point Likert scale (0 = never, 1 = sometimes, 2 = often, 3 = always) (41). The total sum of scores (range 0–63) was used in the analyses.

### 2.7 Fiber and food intake

Prior to the study, fiber intake was measured with the validated FiberScreen tool. This tool is a short screening questionnaire, consisting of 18 items, and suitable for ranking the fiber intake of adults (42). Fiber intake was used to stratify the participants over the two intervention groups. Food intake was measured at t = −3, 0, and 3 weeks using a Smartphone-based dietary assessment Tool (Traqq) (43). Participants recorded their food intake for 2 subsequent days at all three timepoints. Energy, protein, fat, carbohydrate, fiber, and alcohol intake were monitored as potential confounding variables.

### 2.8 Statistical analysis

#### 2.8.1 Per protocol analysis and statistical significance

After the intervention, a blind data review meeting was held, to determine which participants had major protocol violations and should be excluded from PP analysis. Participants were excluded from the PP population, using the predefined criteria: 1. Less than 80% of supplements were used, 2. Antibiotics were used throughout the intervention or, 3. Pre-, pro- or synbiotics (outside the study product) were used during the study period. For the microbiota analyses participants were further excluded from the PP analysis in case stool samples at t = 0 or t = 3 were missing. For the tertiary outcomes, participants were excluded from the PP analyses in case <80% of the questionnaires (per questionnaire) were filled in. Analyses were performed on the PP population only.

All analyses were within group analyses. For the total number of comparisons being performed, both actual *p*-values, as well as adjusted *p*-values based on the Benjamini-Hochberg false discovery rate (FDR) control method (44), were calculated. A two-sided significance level of α = 0.05 was used. For the analysis of the family *Bifidobacteriaceae* and the genus *Bifidobacterium* no multiple testing correction was applied as it was hypothesized prior to the study and documented in the statistical analysis plan that these would increase due to the intervention. These taxa were analyzed one-sided with a significance level of α = 0.05. All analyses were performed using R version 4.3.0 (45, 46).

Changes in the relative abundances of *Bifidobacterium* (primary outcome) and other taxa between the control and the intervention period were assessed using generalized linear mixed models (LMMs) using the lme4 R package (47) and visualized using ggplot2 (48). The study participants were included as a random effect and the time point as a fixed effect. A variable coding for the intervention period was included to indicate whether the observation was made pre or post intervention. The covariates fiber intake at screening, BMI, age, fiber intake during the study and baseline value of the outcome parameter were included as fixed effects.

Overall effects of the intervention on fecal microbial composition (beta-diversity) were determined using the Bray–Curtis dissimilarity calculated with the Vegan package (49). Statistical significance and the proportion of microbial composition variance explained by time and covariates (fiber intake at screening, BMI, age, and fiber intake during the study) were obtained using PERMANOVA using the GUniFrac package (50). To determine the effect of the intervention on α-diversity, Shannon diversity and Richness were calculated at each of the three time points using the Vegan package. LMMs were used to evaluate changes in α-diversity metrics between control and interventional periods.

#### 2.8.2 Statistical analysis of tertiary outcomes

The stool frequency assessment and the summed scores for each of the GI comfort, Sleep quality, and DASS-21 questionnaires were analyzed using LMMs like the microbiota analyses. In all the analyses, time, period, and covariates (fiber intake at screening, BMI, age, and fiber intake during the study) were included as fixed effects, and subject as a random effect.

#### 2.8.3 Subgroup analyses

Exploratory subgroup analyses were performed on responders/non responders, for the relative abundance of *Bifidobacterium*, as indicated in the statistical analysis plan. Responders to GOS intervention were defined as participants for which the increase of the relative abundance of *Bifidobacterium* in the intervention period (between t = 0 and t = 3) was larger than the absolute difference in relative abundance of Bifidobacterium between the start (t = −3) and end (t = 0) of the control period.

Hierarchical clustering of principal components (HCPC), as previously described (51), was used as a non-supervised approach to group participants based on questionnaire responses at baseline (t = 0), subject characteristics such as age and BMI, responder status, microbiome composition at baseline, diet (being on a specific diet, taking supplements), fiber intake at screening and at baseline, and nutrient intake at baseline. The data was centered and scaled to make comparison of data from various sources possible. This approach allows for the discovery of relevant subgroups in the cohort by statistically identifying any associations of study variables to subject clusters.

## 3 Results

### 3.1 Study flow and participants

**Figure 1:**
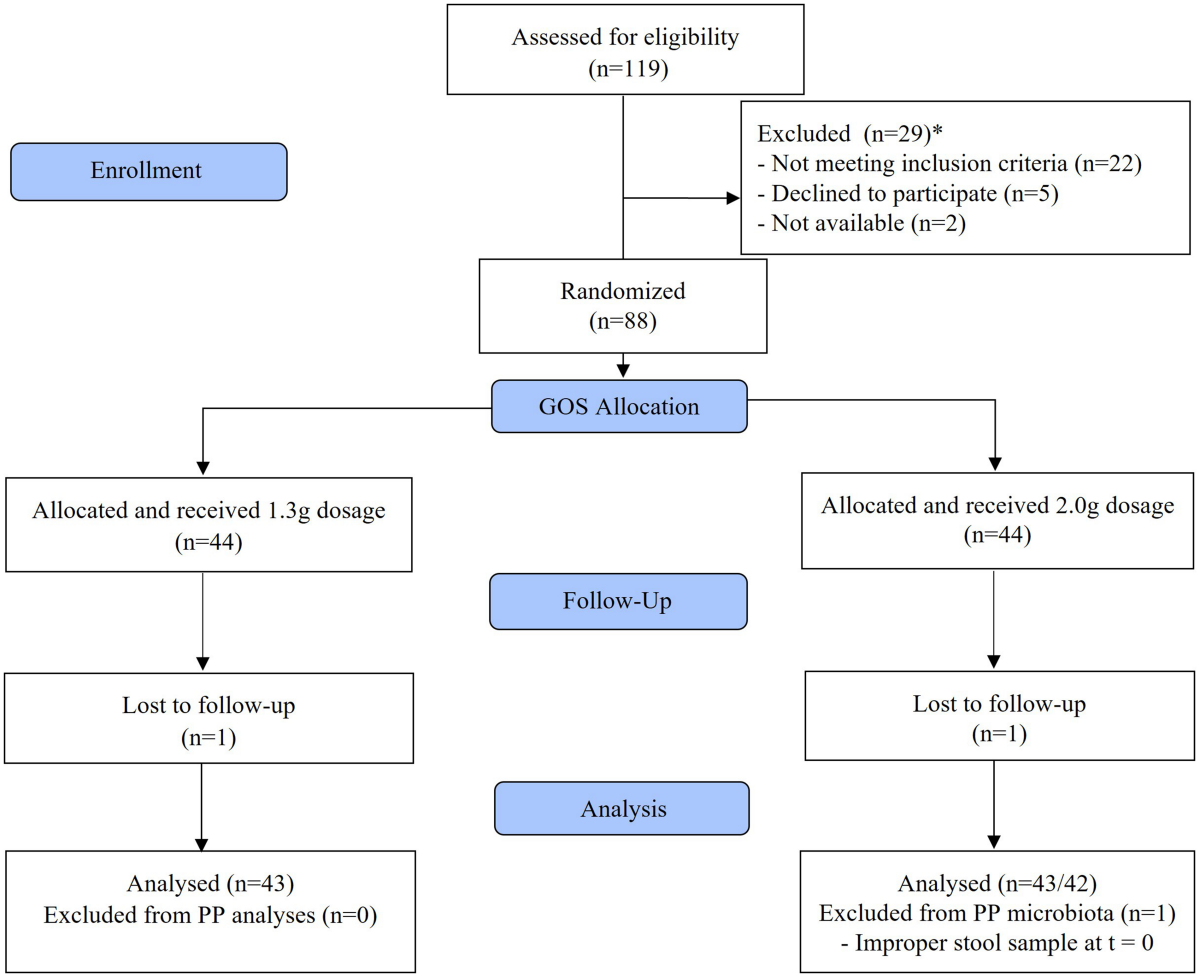
Flow diagram of study participants.

In total 88 women were included in the study. Compliance to study products was high (on average 97.8% of the study products were consumed), the participants tolerated the study product well, and no serious adverse events were reported during the study. Eighty-six participants completed the study. In both intervention groups, one subject dropped out, one because of a required antibiotic treatment, another due to personal reasons. One subject of the 2.0 g GOS group was excluded from the PP analysis of the microbiota analyses because the stool sample of t = 0 was taken after starting the intervention. This subject was included in the PP analyses of the questionnaires. The flow diagram for participants is shown in Figure 1.

The groups were comparable with respect to the distribution of participants consuming vitamin or mineral supplements, a specific diet, and alcohol (Table 1).

**Table 1:** Baseline characteristics of participants per intervention group, included in the PP analysis.

	Microbiota and tertiary outcome PP group 1.3 g GOS (*n* = 43)	Microbiota PP group 2.0 g GOS (*n* = 42)	Tertiary outcome PP group 2.0 g GOS (*n* = 43)
BMI (kg/m^2^)	24.2 ± 2.8	23.6 ± 2.8	23.6 ± 2.8
Age (year)	57.4 ± 7.7	56.6 ± 7.1	56.8 ± 7.2
Fiber intake at screening (g/day)	21.6 ± 5.10	21.9 ± 5.7	22.1 ± 5.8
Nutritional supplement users^*^ (*n*)	20	19	20
Specific diet^**^ (*n*)	6	5	5
Alcohol users (*n*)	28	24	24

BMI, age, and fiber intake are mean ± SD.

* Vitamin and mineral supplements.

** Vegan, vegetarian, flexitarian, or gluten free diet.

### 3.2 Effects of low dosages of GOS on fecal microbiota composition

The effects of 1.3 and 2.0 g of GOS on *Bifidobacterium* relative abundance (primary outcome) and general microbiota composition (secondary outcome) were determined using shotgun metagenomics sequencing. The sequencing data were of high quality with 1.9 to 7.8 million sequencing reads per sample per direction (mean: 5.8 million, SD: 0.79 million). Sequencing reads had a Q score of 35.3 (SD: 0.32) and a length of 144.7 bp (SD: 3.6 bp). After quality controls, the number of reads that could be successfully classified using Kraken2 per sample ranged from 0.28 to 3.8 million (mean: 1.34 million, SD: 0.63 million). A multi-dimensional scaling plot of Bray-Curtis analysis performed on all samples showed a scattered sample cloud without any outliers and all samples were included in the analyses (Supplementary Figure 1).

**Figure 2:**
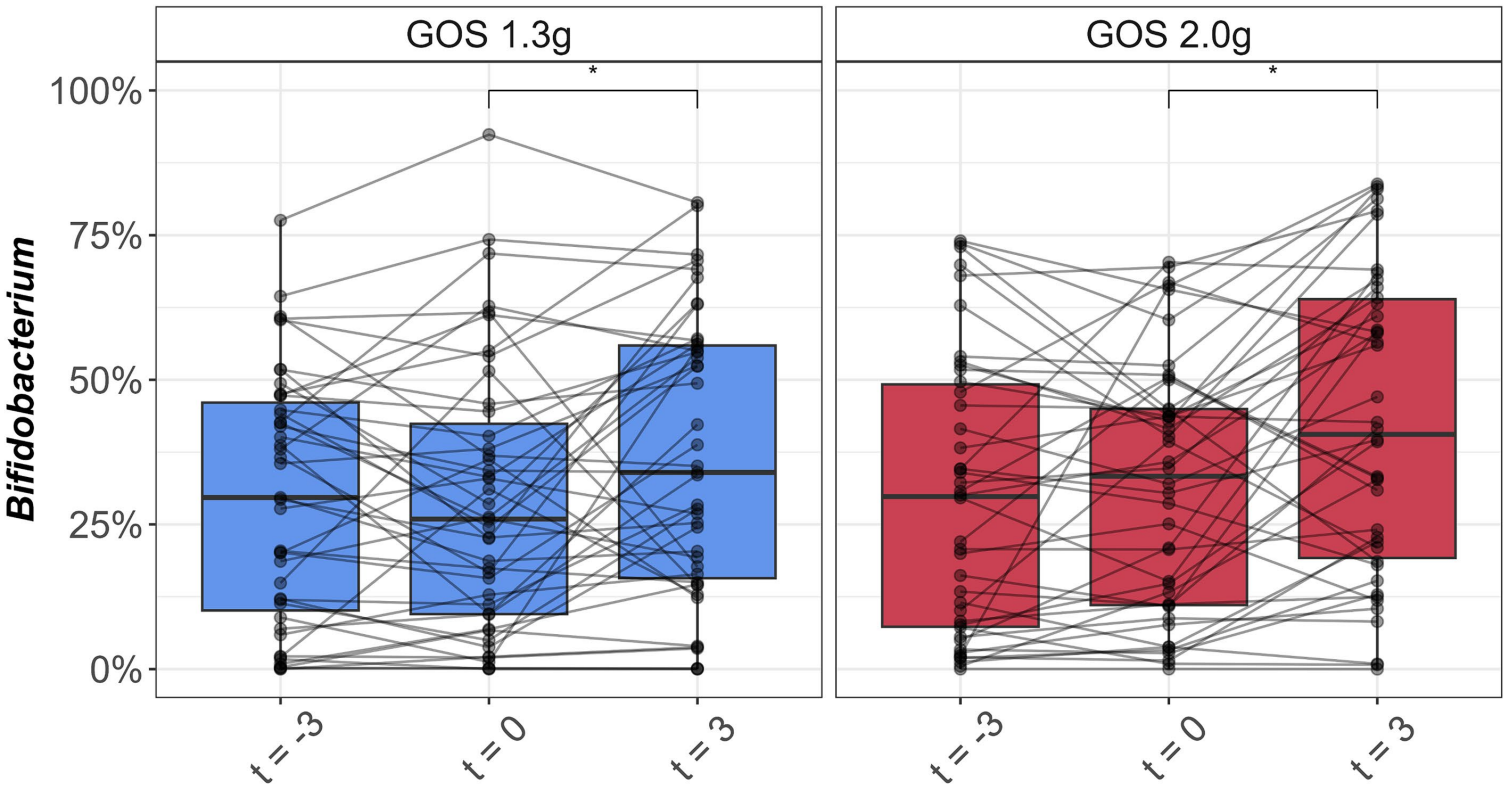
Boxplots of relative abundance of *Bifidobacterium* (%) in fecal samples taken at the start (t = −3) and end (t = 0) of the 3-week control period, and at the end (t = 3) of the 3-week intervention period with 1.3 g GOS (left panel; *n* = 43) and 2.0 g GOS (right panel; *n* = 42) per day. *A significant increase of *Bifidobacterium* was observed after 3 weeks of GOS supplementation in both 1.3 and 2.0 g groups (*p* < 0.01).

Average levels of *Bifidobacterium* in the control period were 29.5% (SD: 21.6%; t = −3) and 29.0% (SD: 23.3%; t = 0) for the 1.3 g GOS group and 29.6% (SD: 24.0%; t = −3) and 31.5% (SD: 21.9%; t = 0) for the 2.0 g GOS group. The levels were significantly higher after 3 weeks of daily intervention with both 1.3 g [36.8% (SD: 24.2%); *p* < 0.01] and 2.0 g GOS [42.3% (SD: 26.2%); *p* < 0.01; Figure 2].

**Figure 3:**
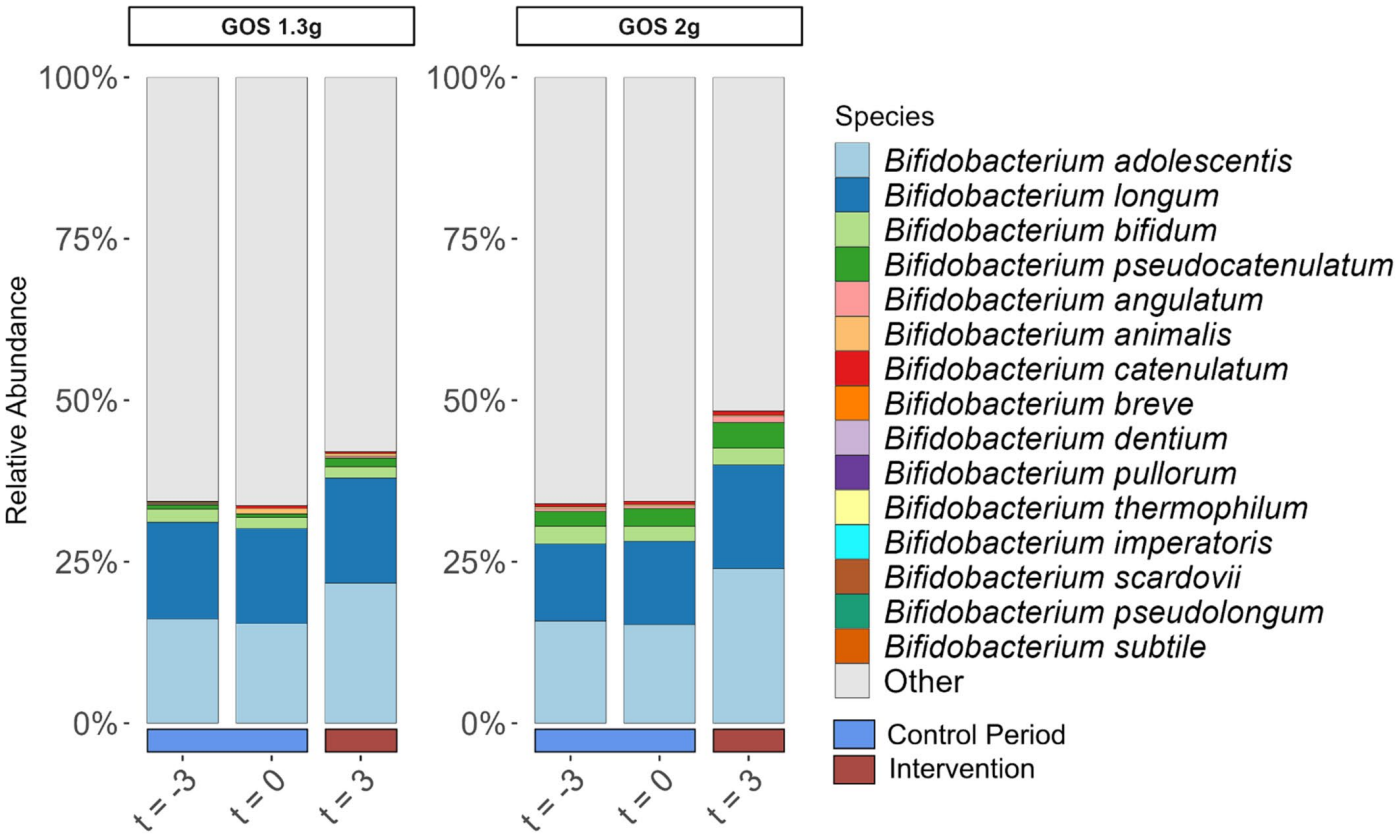
Relative abundance of *Bifidobacterium* species (% of total community) in fecal samples taken at the start (t = −3) and end (t = 0) of the 3-week control period, and at the end (t = 3) of the 3-week intervention period with 1.3 g GOS (left panel; *n* = 43) and 2.0 g GOS (right panel; *n* = 42) per day. *B. adolescentis* significantly increased due to intervention within both groups of 1.3 g (*p* < 0.01) and 2.0 g GOS (*p* = 0.03).

*Bifidobacterium adolescentis* and *Bifidobacterium longum* were the dominant bifidobacterial species pre- and post-intervention (Figure 3). Of these two species, only *B. adolescentis* significantly increased due to intervention within both groups of 1.3 g (*p* < 0.01) and 2.0 g GOS (*p* = 0.03).

**Figure 4:**
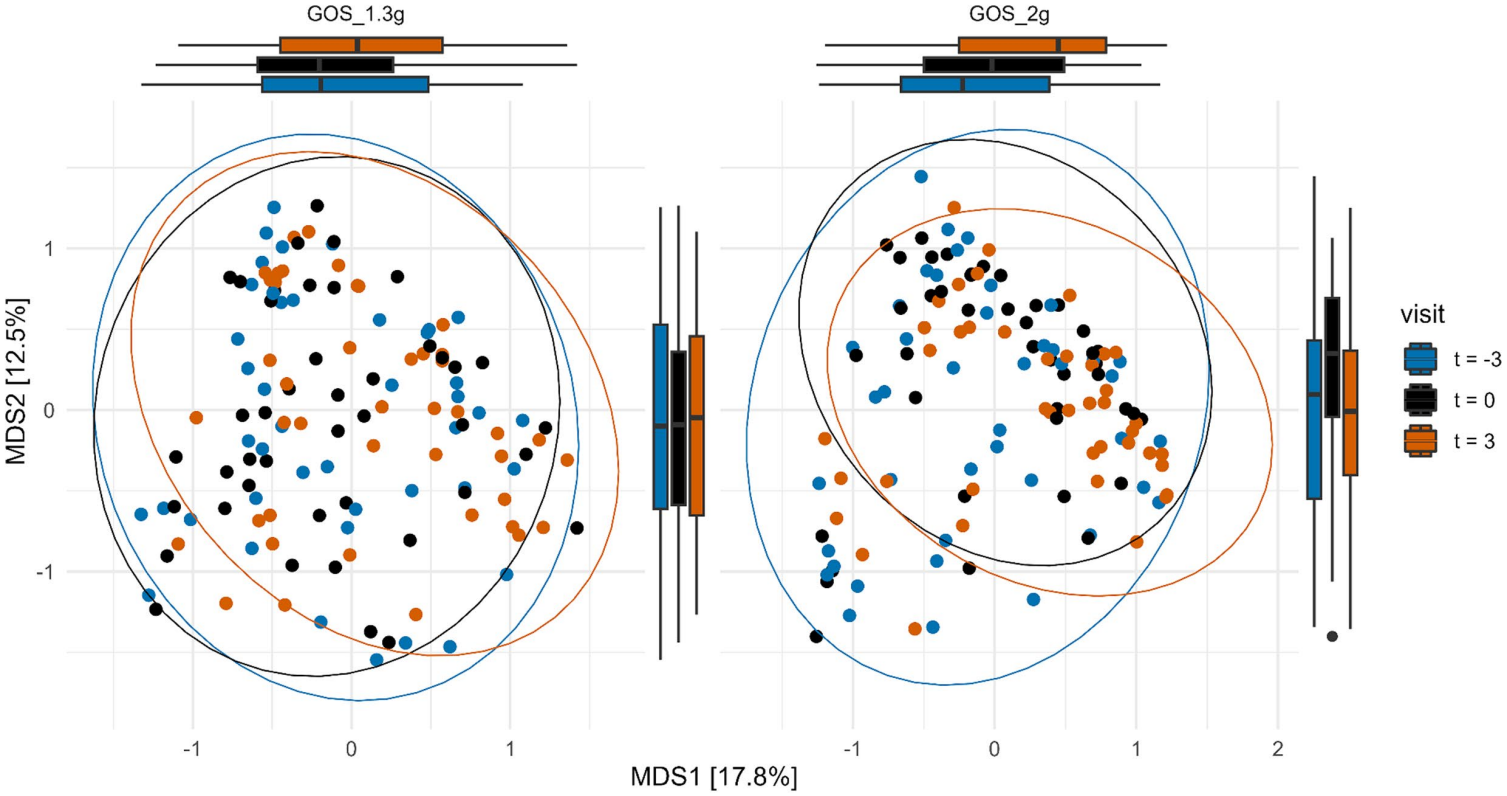
Multi-dimensional scaling (MDS) plot of Bray-Curtis distances of samples taken at the start (t = −3) and end of the 3-week control period (t = 0), and at the end (t = 3) of the 3-week intervention with 1.3 g GOS (left panel; *n* = 43) and 2.0 g GOS (right panel; *n* = 42). Overall microbiota composition was significantly different (*p* < 0.01) after intervention (t = 3) compared to the control period (t = −3 and t = 0) for the 2.0 g GOS group based on PERMANOVA analysis.

To determine the effect of the interventions on total microbiota composition, PERMANOVA analysis of Bray-Curtis distances was performed. A significant (*p* < 0.01) difference in fecal microbiota composition between the samples taken at the three different time points in the 2.0 g GOS group, but not within the 1.3 g GOS group (*p* = 0.13) was revealed (Figure 4).

Species richness was similar for both intervention groups and not influenced by GOS intervention. Shannon diversity, which considers both species richness and evenness, decreased during the intervention with 2.0 g GOS (*p* < 0.01; Supplementary Figure 2).

The microbiota profiles of individual participants at genus level at the different time points are shown in Supplementary Figure 3. Differences over time in the relative abundances of taxa at each phylogenetic level (phylum, order, family, genus, and species) were assessed using LMMs. After correction for multiple testing (~1,000 species tested), no taxa were significantly different after the intervention period as compared to the control period.

### 3.3 Effect of low dosages of GOS on GI comfort, stool frequency, sleep quality, and mental wellbeing

**Figure 5:**
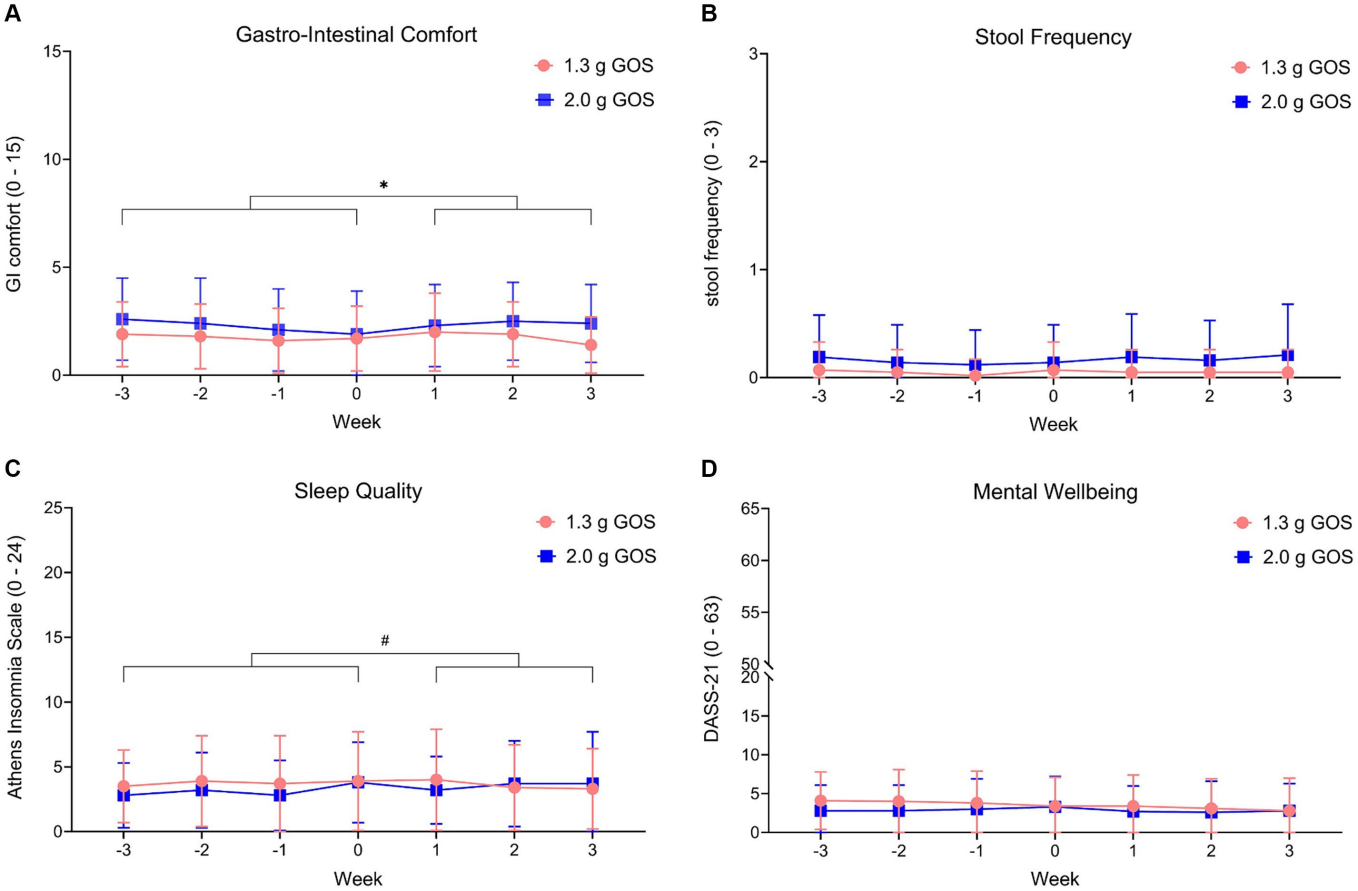
Wellbeing parameters gastro-intestinal comfort **(A)**, stool frequency **(B)**, sleep quality **(C)** and mental wellbeing **(D)**, within the 1.3 g GOS group and the 2.0 g GOS group. Week −3 to week 0 is control period; week 1 to week 3 daily GOS supplement is used. Scoring ranged from 0 to 15 for GI comfort, from 0 to 24 for sleep quality and from 0 to 63 for mental wellbeing. Higher scores indicate more complaints. Stool frequency ranges from 0 (>5 times a week) to 3 (<1 time a week). *A significant difference (*p* < 0.01) between intervention and control period was observed for gastro-intestinal comfort, in the 2.0 g GOS group only. # A trend (*p* = 0.09) was observed for sleep quality in the 1.3 g GOS group only.

GI comfort scoring throughout the study, showed that participants of the 2.0 g GOS group experienced somewhat more gut complaints in the intervention period compared to the control period (*p* < 0.01). In this group, on average, total symptom score for GI comfort slightly decreased in the control period (“more comfort”), then slightly increased during the first 2 weeks of the intervention to a level similar to the start of the control period and reduced again during the 3^rd^ week of the intervention (Figure 5A). No significant gut comfort differences were observed between the control and intervention period for the 1.3 g GOS group. Stool frequency and mental wellbeing were not significantly different between the control and intervention period for either intervention groups (Figures 5B,D). For sleep quality, there was a trend (*p* = 0.09) towards reduced sleep complaints during the intervention period in the 1.3 g GOS group. Of note, the 2.0 g GOS group had a slightly lower average baseline score for sleep quality (less complaints) as compared to the 1.3 g GOS group (Figure 5C).

### 3.4 Responder analysis

#### 3.4.1 *Bifidobacterium* species analysis

**Figure 6:**
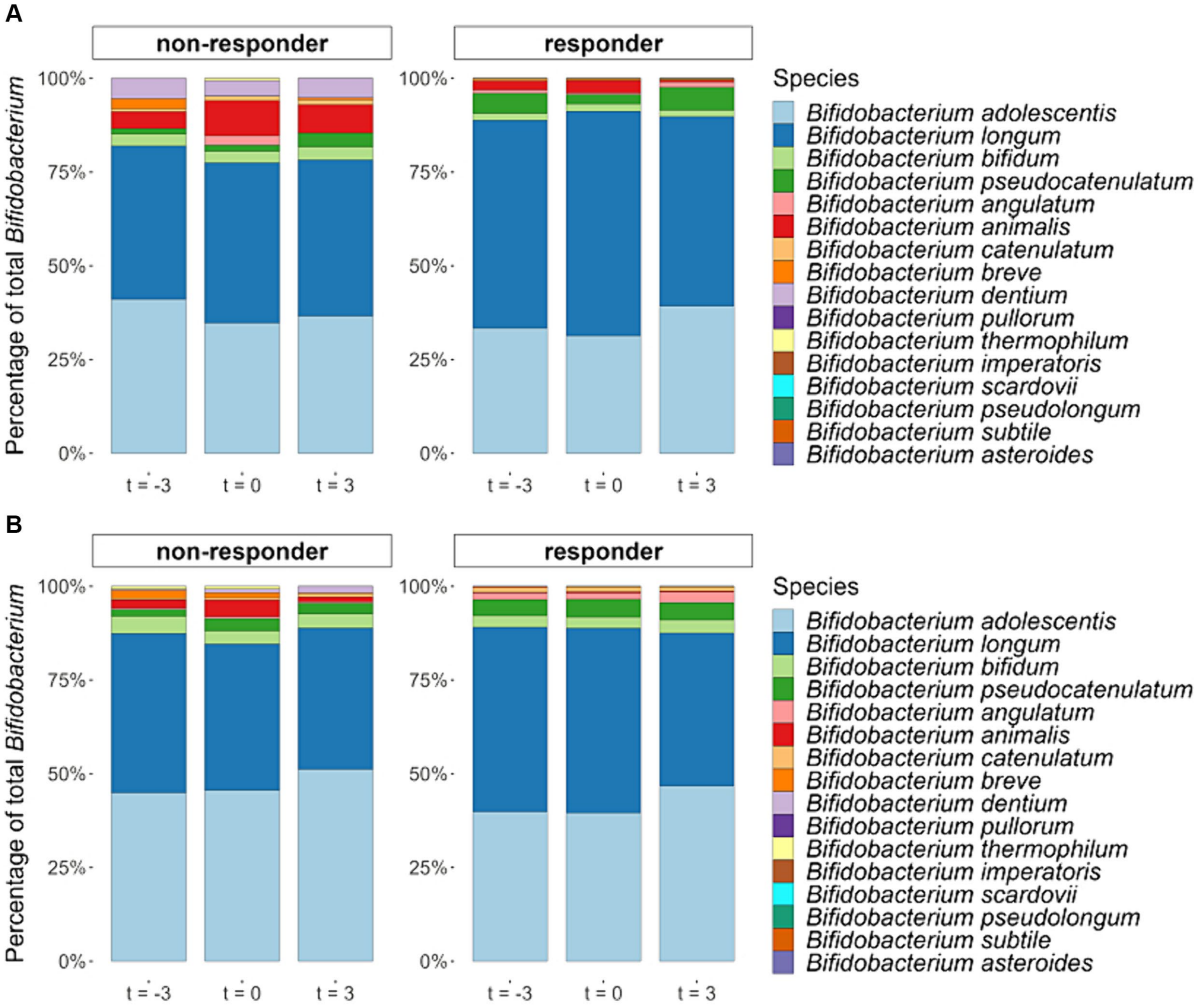
Averaged relative abundances of *Bifidobacterium* species (percentage of all detected *Bifidobacterium* species together) in fecal samples taken at the start (t = −3) and end (t = 0) of the 3-week control period and at the end (t = 3) of the 3-week intervention period with either 1.3 g GOS **(A)** or 2.0 g GOS **(B)** per day for responders (*n* = 43) and non-responders (*n* = 42). In the responders, *B. longum* significantly decreased in both intervention arms in the 1.3 (*p* < 0.01) and 2.0 g GOS group (*p* = 0.03).

Although on average, the relative abundance of fecal *Bifidobacterium* significantly increased during the interventions, this was not observed for all participants. Between the start and the end of the control period, in which the participants did not consume any GOS supplements, the relative abundances of *Bifidobacterium* fluctuated. When comparing the increase of *Bifidobacterium* in the intervention period with the absolute variation of *Bifidobacterium* in the control period, 20 of the 43 participants of the 1.3 g GOS group, and 23 of the 42 participants of the 2.0 g GOS group were classified as responders. Baseline microbiota composition of both intervention groups combined was significantly different between responders and non-responders based on PERMANOVA analysis of Bray-Curtis distances (*p* = 0.04; Supplementary Figure 4). Statistical analysis further showed that there was a trend towards lower *Bifidobacterium* at baseline for responders compared to non-responders (*p* = 0.10; Supplementary Figure 5). During the intervention period, the distribution of *Bifidobacterium* species changed in the responders, especially for the two dominant species *B. adolescentis* and *B. longum*. The relative part of *B. adolescentis* increased (although this was statistically not significant) while that of *B. longum* significantly decreased in both intervention arms (*p* < 0.01 and *p* = 0.03, for 1.3 and 2.0 g respectively). In the non-responders, no statistically significant effect of the intervention on the distribution of *B. longum* and *B. adolescentis* was found (Figure 6).

#### 3.4.2 GI comfort, stool frequency, sleep quality, and mental wellbeing

There was no statistically significant effect of the intervention on GI comfort for the responders in either of the treatment groups, probably as gut complaints were already low at baseline (Supplementary Figure 6A). Stool frequency and mental wellbeing were also not significantly different between the two periods for both intervention arms, within the responder analysis (Supplementary Figures 6B,D). For the responders in the 1.3 g GOS intervention group, like the total study population, sleep quality tended to be improved in the intervention period compared to the control period (*p* = 0.07). This was not observed for the responders in the 2.0 g GOS group, that had lower scores (“better sleep quality”) at baseline than the responders in the 1.3 g GOS group (Supplementary Figure 6C).

### 3.5 Hierarchical clustering of principal components

HCPC was used to cluster participants according to their similarity across many measured parameters. To assess the natural variation across the cohort, we applied HCPC on responder status and baseline (t = 0) measurements of microbiome species abundance, dietary intake and habits, and questionnaire outcomes. This resulted in four main clusters (Supplementary Table 1).

The most important characteristics that were statistically associated with the four clusters are as follows (all reported statistics were *p* < 0.05): 85% of the participants in cluster 1 (*n* = 20 in total) were non-responders. The participants in this cluster were, on average, younger (cluster average 48.5 years old vs. an average of 57 years in the total study population). They had on average a 2.8 times lower baseline relative abundance of *Akkermansia muciniphila* (4.4 vs. 12%) but 2.3 times higher baseline relative abundances of *Bifidobacterium* sp., possibly contributing to their non-responder status.

Of the participants in cluster 2 (*n* = 37 in total), 73% were responders vs. 49% in the total study population. All participants in this cluster indicated they did not follow a diet, such as a vegetarian, vegan, or gluten free (13% of the total study population reported being on a diet). Furthermore, compared to the average of the total study population, baseline *A. muciniphila* relative abundance was 1.7 times lower (7.1 vs. 12%). Participants in cluster 2 were on average older than the average (59.4 years old compared to 57 years for the total study population).

Responder status was not statistically associated with cluster 3, meaning that the 14 participants of this cluster were a mixture of responders and non-responders. Seventy-nine % of participants in this cluster took supplements vs. 46% in the total study population. In this cluster, relative abundances of *B. longum and B. adolescentis* were on average 2.8 times lower than the average across the cohort, most likely explained by a higher number of responders (*n* = 9) vs. non-responders (*n* = 5), although not statistical significantly different. *A. muciniphila* relative abundances were highest in this cluster with an average relative abundance of 39% compared to 12% in the rest of the cohort.

Like cluster 3, cluster 4 (*n* = 14 in total) was a mixture of responders and non-responders. Fifty-seven % of the participants in this cluster indicated following a diet, and supplement usage was relatively high (79% of participants taking supplements compared to 46% across the total study population). Participants of cluster 4 were the oldest of the cohort, being on average 64 years old vs. the cohort average of 57 years. Finally, their intake of fiber, non-heme iron, and folate was higher than average, but heme-iron intake was below average.

## 4 Discussion

To our knowledge, this is the first study in healthy women that thoroughly examined the impact of low dosages of prebiotic GOS on gut microbiota composition using state-of-the-art microbiota analyses techniques. The study shows that low dosages of GOS significantly increased fecal levels of *Bifidobacterium*. Additionally, a global shift in the microbiota composition was observed when a 2.0 g dose of GOS was consumed daily. This indicates that GOS not only have the ability to selectively enrich beneficial microorganisms like *Bifidobacterium* sp. but might also result in broader shifts in the community structure.

The bifidogenic effects of GOS have been demonstrated in several studies but mainly for higher dosages of GOS (13–23). GOS dosages of 1.3 and 2.0 g, as studied in the current trial, are low enough for application as supplements in the form of capsules or tablets. In the current study, women between 42 and 70 years were included, to be able to compare the results with outcomes of previous studies that were also only conducted in women (15, 17, 19); those studies showed bifidogenic effects with higher daily dosages of GOS (5.5–15 g). Another reason to include women only is that supplement use, especially for gut and digestive health, is generally higher in women than in men and increases with age. An age range of 40–70 years was chosen because in general bifidobacteria tend to decrease with age in a healthy population, specifically after the age of 40 (30, 52). People of 40 years and older will therefore benefit most from supplements that increase bifidobacteria. In people above 70, there is a higher chance that other conditions or diseases come into play that may impact on the study results (53); we therefore limited the age range to 70 years. Since our study found similar bifidogenic effects of GOS compared to studies with men only (35, 36) and a study in a general healthy population (13), no large differences in bifidogenic effects of low dosages of GOS are expected between men and women. In the current study, the reported baseline level of *Bifidobacterium* is higher than observed in recent studies using similar metagenomic techniques and bioinformatic workflows. This is likely a result of the high Kraken2 confidence parameter, which was set to 0.9. This confidence parameter was used to reduce the number of false positives when classifying sequencing reads (see materials and methods) and resulting in an increased relative abundance of *Bifidobacterium* (about 15%, data not shown) compared to the default confidence setting of 0.1. We applied the high confidence setting to generate a more accurate and reliable classification of sequencing reads on the species level, as we aimed to focus on potential changes in *Bifidobacterium* species.

In the current study, the two most abundant bifidobacterial species at baseline constituted *B. adolescentis* and *B. longum*, which is typical for healthy adults (31). Although *in vitro* data show that single strains of both *B. longum* and *B. adolescentis* grow very well on GOS (54), our *in vivo* data show that the significant increase in *Bifidobacterium* mainly resulted from the stimulation of *B. adolescentis*. In the responders of the study, the ratio between *B. adolescentis* and *B. longum* increased due to the intervention. As far as we know, a differential effect of GOS on *Bifidobacterium* species in the adult gut has not been demonstrated before. Further research is required to get insight into the physiological relevance of this observation.

Species richness, i.e., the number of distinct species in a sample, of the microbiota was not influenced by the GOS interventions. A significant decrease in Shannon diversity was observed during the intervention period for the 2.0 g GOS group only. This has been observed before (22). As Shannon diversity not only considers species richness, but also species evenness, the result of a decreasing diversity in response to 2.0 g GOS is to be expected with a prebiotic intervention, as it selectively enriches specific microbiome members. Thus, specific enrichment of species, such as *Bifidobacterium* in our intervention, might decrease (Shannon) α-diversity (55).

GOS has previously been shown to positively influence gut comfort (20, 26, 27), mental wellbeing (17) and sleep (56). Except for a trend towards improved sleep in the 1.3 g GOS group, other significant positive effects were not revealed in the current study. Most likely as the study was not powered for these outcome parameters, in contrast to the referred studies. Furthermore, the participants in the current study already scored low at baseline on problems related to gut comfort, mental wellbeing, and sleep. Therefore, it is difficult to improve on these parameters. Gut complaints slightly increased in the 2.0 g GOS group during the first 2 weeks of the intervention but returned to normal levels towards the end of the 3^rd^ week of the intervention. Potentially, the microbiota adapted in this period to efficiently ferment GOS, as has been observed before (57).

The microbiome is complex, being influenced by both environmental and genetic factors. This makes it difficult to control when recruiting subjects for a clinical trial. To better understand the natural variation in the cohort, and how this variation could be associated to responder status, we performed a HCPC analysis. This technique allowed us to identify natural groupings of participants based on responder status and all measurements taken prior to the GOS intervention (baseline t = 0). The resulting clustering grouped participants into four main clusters. Clusters 1 and 2 were primarily associated with responders and non-responders respectively, and contained relatively young and older participants of the cohort, respectively. We also observed that responders typically had a lower initial relative abundance of *Bifidobacterium* compared to non-responders. Together, results for these two clusters align with what is observed in the literature, where aging is associated with decreasing *Bifidobacterium* levels in the fecal microbiome (30). After *Bifidobacterium*, *Akkermansia* was the most abundant genus detected in the fecal samples in our study with *A. muciniphila* as the only species. Both cluster 1, mainly non-responders, and 2, mainly responders, were associated with a lower relative abundance of *A. muciniphila* at baseline compared to the total study population whereas cluster 3, a mixture of responders and non-responders, showed the highest levels of *A. muciniphila* at baseline. This indicates that baseline levels of *A. muciniphila* are not associated with responder status although differences in initial microbiota composition definitely can play a role in how people respond to dietary interventions, such as pre/pro-biotics and supplements (58, 59). Here, clusters 3 and 4 were a mixture of responders and non-responders, likely due to stronger factors than responder status driving the clustering. Although the subjects could be grouped in 4 separate clusters on basis of their characteristics, no clear associations with responsiveness to the intervention (defined as a bifidogenic effect) could be revealed.

Studies that address the effect of prebiotics typically only compare the microbiota composition at the start and the end of the intervention. A key strength of the present study is that the intervention period was preceded by a control period, without any intervention taking place, to get insight in the natural variation of the gut microbiota composition over a period equal to the intervention period. By taking both the microbiota composition at the start and the end of the control period into account, more accurate insight into the intervention’s effect, as compared to the natural variation, on gut microbiota composition was obtained. Another strength is that this study is a within-subject study, with less variation in microbiota compared to between-subject data. Furthermore, it is known that BMI, age, and fiber intake affect microbiota composition (60–62) and therefore participants were stratified for these parameters. The study was performed without a placebo group. For the microbiota related outcomes, we expect that a control period is a better alternative because of the considerable interindividual variation in gut microbiota composition. However, for obtaining conclusive tertiary outcomes, which are subjective measures, it might have been more accurate to include a placebo group. Also, for the microbiota analysis, even more conclusive evidence might be obtained when including a placebo group in addition to the control period.

In this study, the focus was in the first instance to investigate whether low dosages of GOS have a bifidogenic effect. A next step would be to further explore the health benefits related to the increase in relative abundance of bifidobacteria, in a study designed explicitly for this purpose. Future studies could include an extended intervention period and more sampling moments, also after the intervention, to learn when the highest levels of *Bifidobacterium* are reached in time and to learn about the duration of the beneficial impact. In addition, inclusion of *Bifidobacterium* cell count measurement (e.g., via qPCR) would add absolute quantitative insight, and functional analysis of the metagenomes could provide insight in the modulation of microbial metabolic pathways.

In conclusion, this study in healthy women showed significant effects of low dosages of GOS on *Bifidobacterium* relative abundances. This substantiates the beneficial impact of such low dosages of GOS to support a healthy microbiome.

## Data availability statement

The raw sequencing data for this study have been deposited in the European Nucleotide Archive (ENA) under accession number PRJEB78698.

## Ethics statement

The study involving humans was approved by the Medical Ethical Committee of Oost-Nederland. The study was conducted in accordance with the local legislation and institutional requirements. The participants provided their written informed consent to participate in this study.

## Author contributions

EL: Conceptualization, Methodology, Writing – original draft, Writing – review & editing. MS: Conceptualization, Methodology, Writing – review & editing. MB: Data curation, Investigation, Methodology, Resources, Writing – review & editing. EH: Formal analysis, Software, Visualization, Writing – review & editing. GK: Formal analysis, Resources, Visualization, Writing – review & editing. MV-D: Conceptualization, Methodology, Project administration, Resources, Supervision, Writing – review & editing. AN: Conceptualization, Methodology, Writing – review & editing.
