# Supplementary material for: A double-blind intervention trial in healthy women demonstrates the beneficial impact on Bifidobacterium with low dosages of prebiotic galacto-oligosaccharides
Source: Front Nutr. 2024 Aug 19;11:1440319. doi: 10.3389/fnut.2024.1440319 (PMC11366710; doi:10.3389/fnut.2024.1440319)
Supplement: Supplementary file 1 [file Data_Sheet_1.docx]

Supplementary Material

A double-blind intervention trial in healthy women demonstrates the beneficial impact on *Bifidobacterium* with low dosages of prebiotic galacto-oligosaccharides

**Ellen Looijesteijn^1*^, Marieke H. Schoemaker^1^, Maartje van den Belt^2^, Eric R. Hester^3^, Guus A.M. Kortman^3^, Mirre Viskaal-van Dongen^1^, Arjen Nauta^1^**

^1^FrieslandCampina, Amersfoort, The Netherlands

^2^Wageningen Food and Biobased Research, Wageningen University & Research, Wageningen, The Netherlands

^3^NIZO food research B.V., Ede, The Netherlands

*** Correspondence:** Ellen Looijesteijn: ellenlooijesteijn@frieslandcampina.com

# Supplementary Figures and Tables

## Supplementary Figures

**
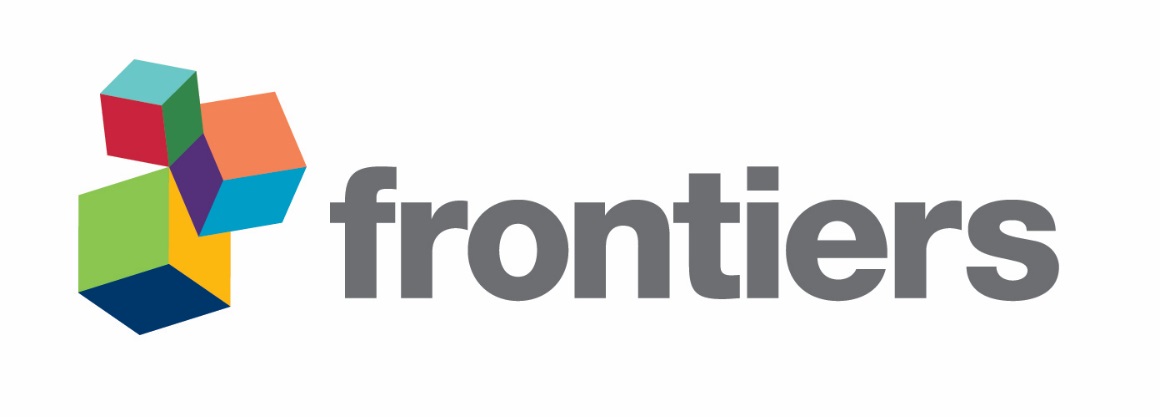
**


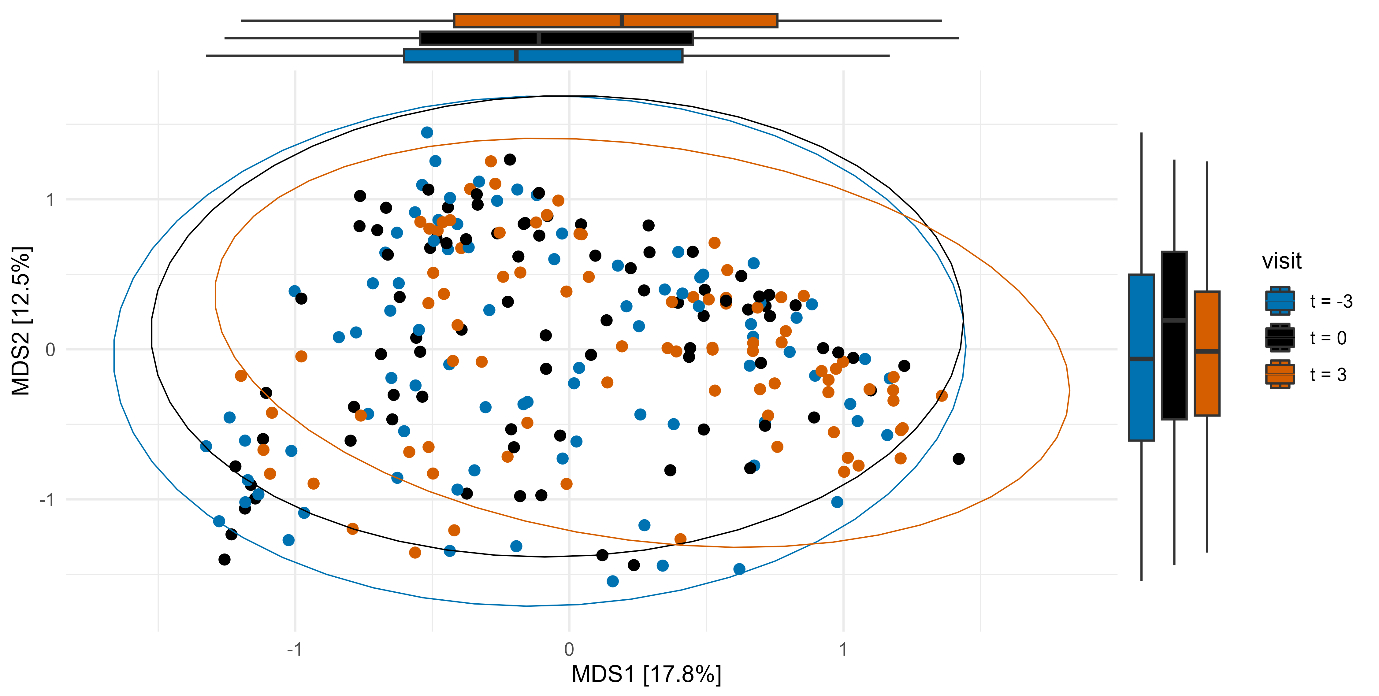


**Supplementary Figure 1.** Multi-dimensional scaling (MDS) plot of Bray-Curtis distances of samples taken at the start (t=-3) and end of the 3-week control period (t=0), and at the end (t=3) of the 3-week intervention, both intervention groups combined (n=85). No outliers were detected.

A


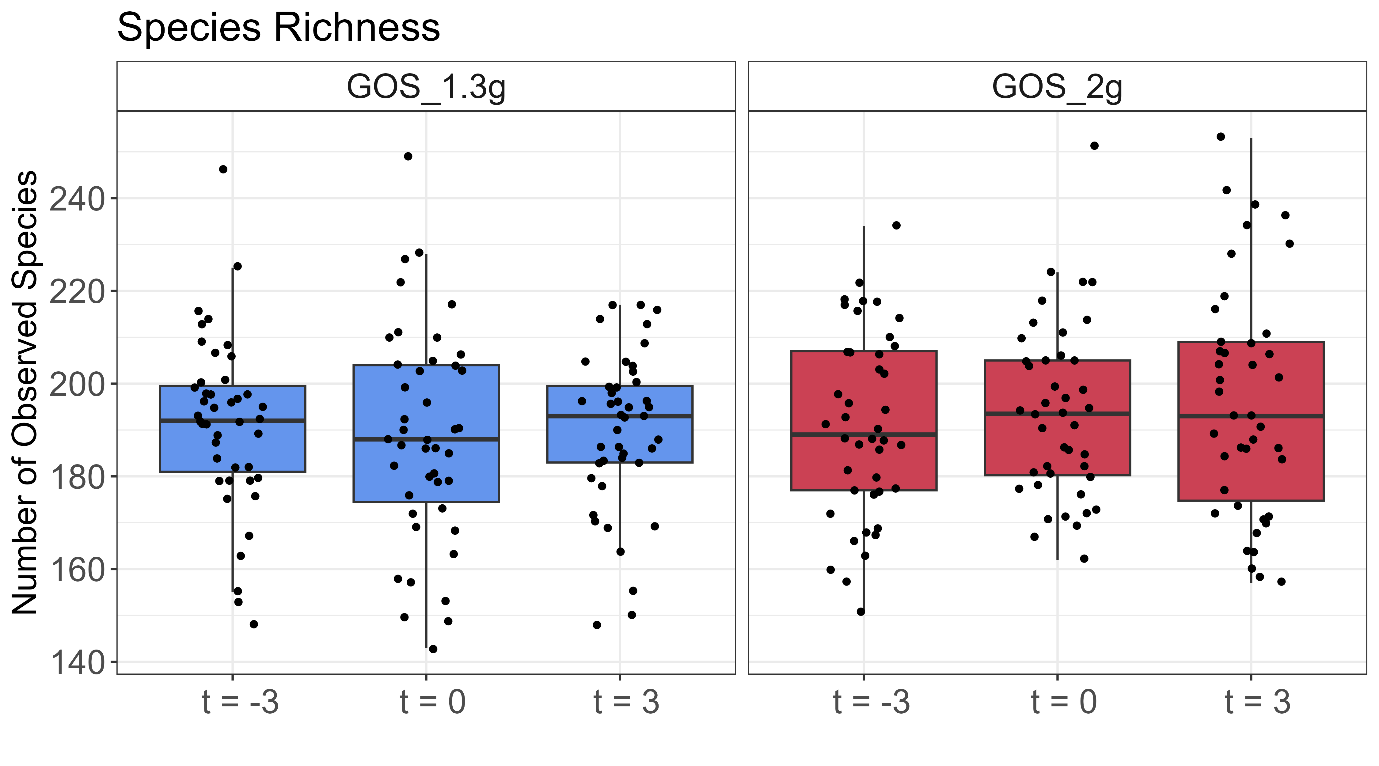


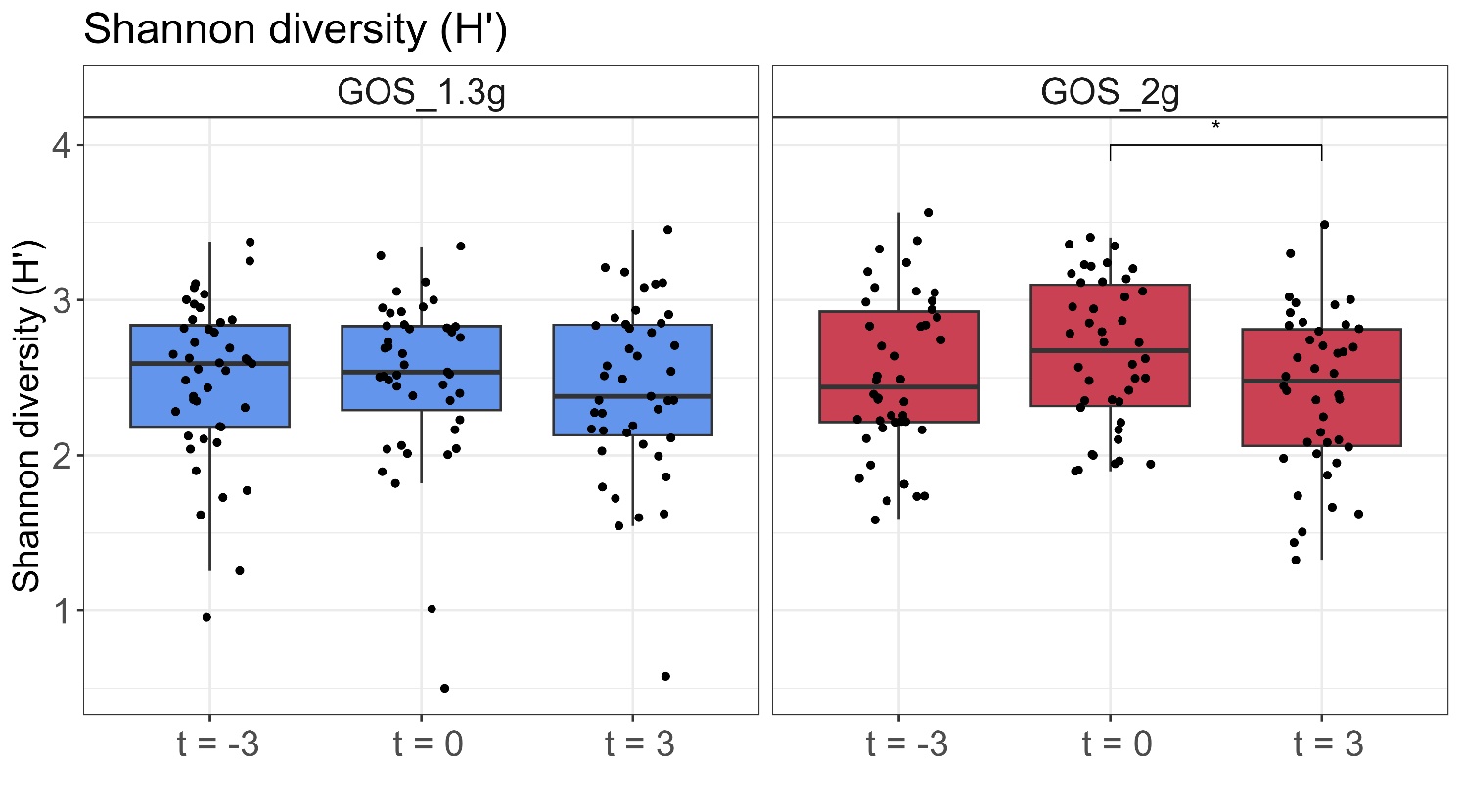


B

**Supplementary Figure 2.** Richness (A) and Shannon α-diversity (B) in fecal samples taken at the start (t=-3) and end (t=0) of the 3-week control period and at the end (t=3) of the 3-week intervention period with 1.3g GOS (left panel; n=43) and 2.0g GOS (right panel; n=42) per day. * Shannon diversity decreased during the intervention with 2.0g GOS (*p*<0.01) compared to baseline (t=0).


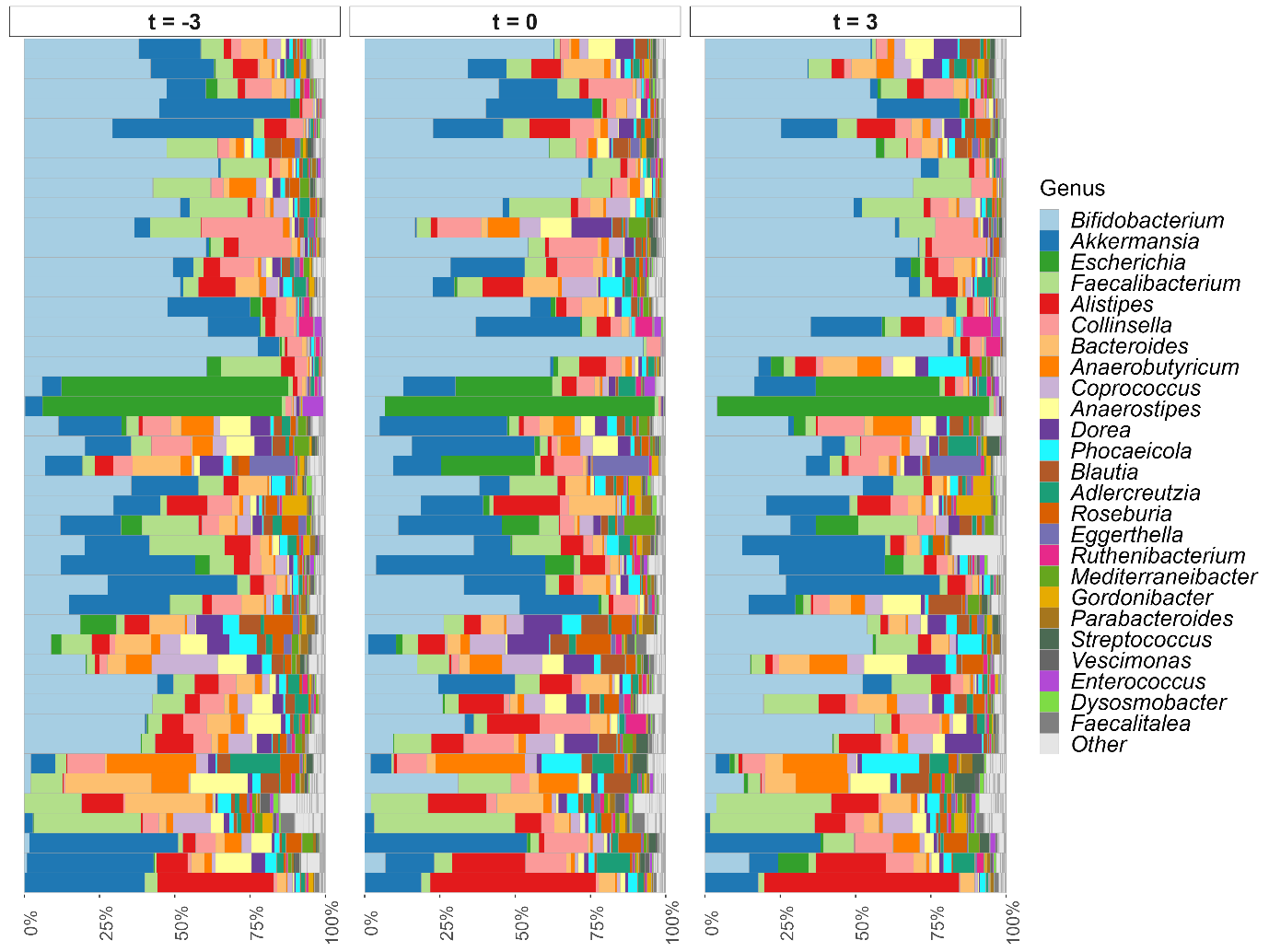


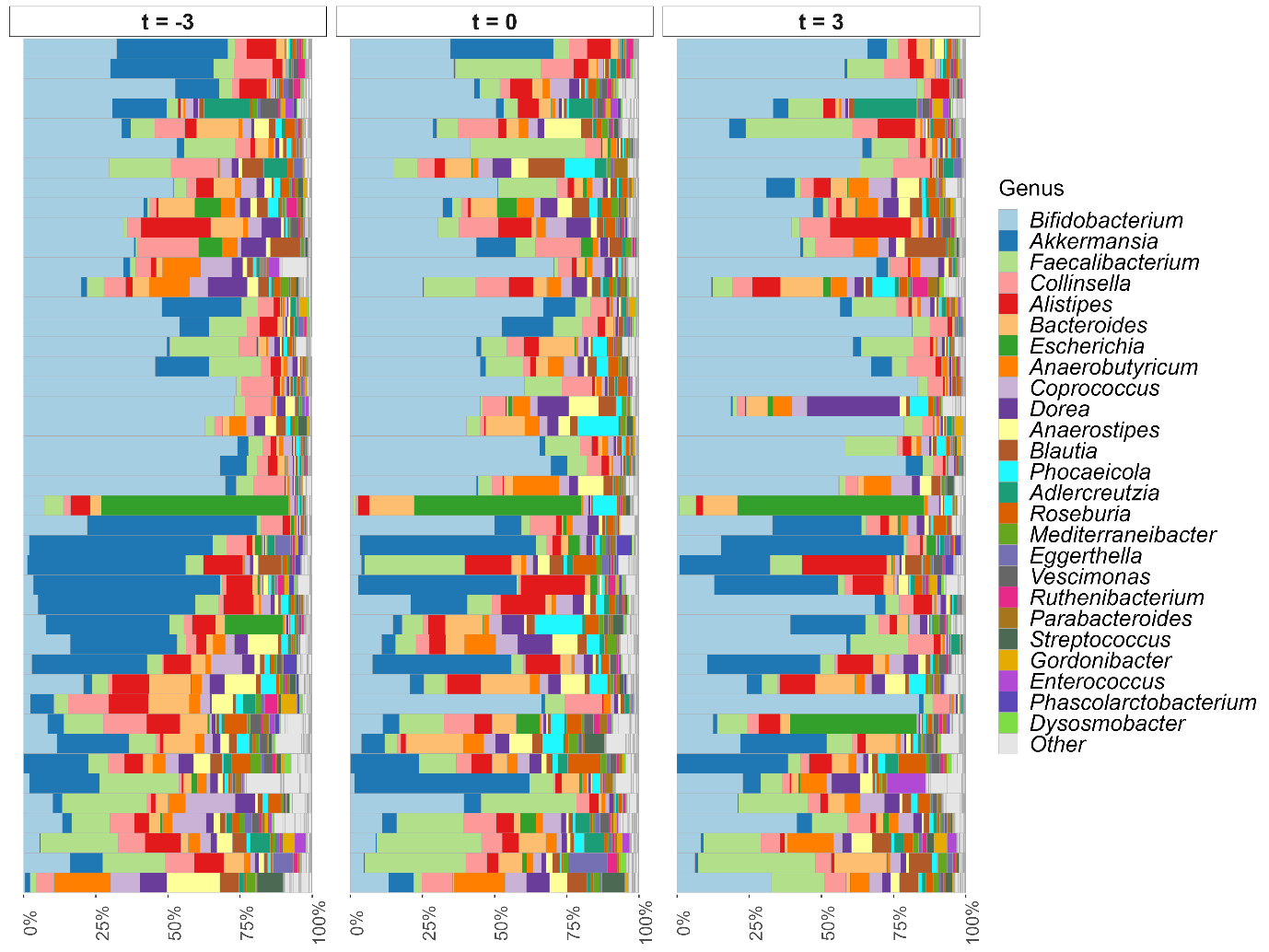


**Supplementary Figure 3.** Relative abundance of bacterial genera in individual fecal samples of participants taken at the start (t=-3) and end (t=0) of the 3-week control period, and at the end (t=3) of the 3-week intervention period with 1.3g GOS (upper panel; n=43) and 2.0g GOS (lower panel; n=42) per day.

**
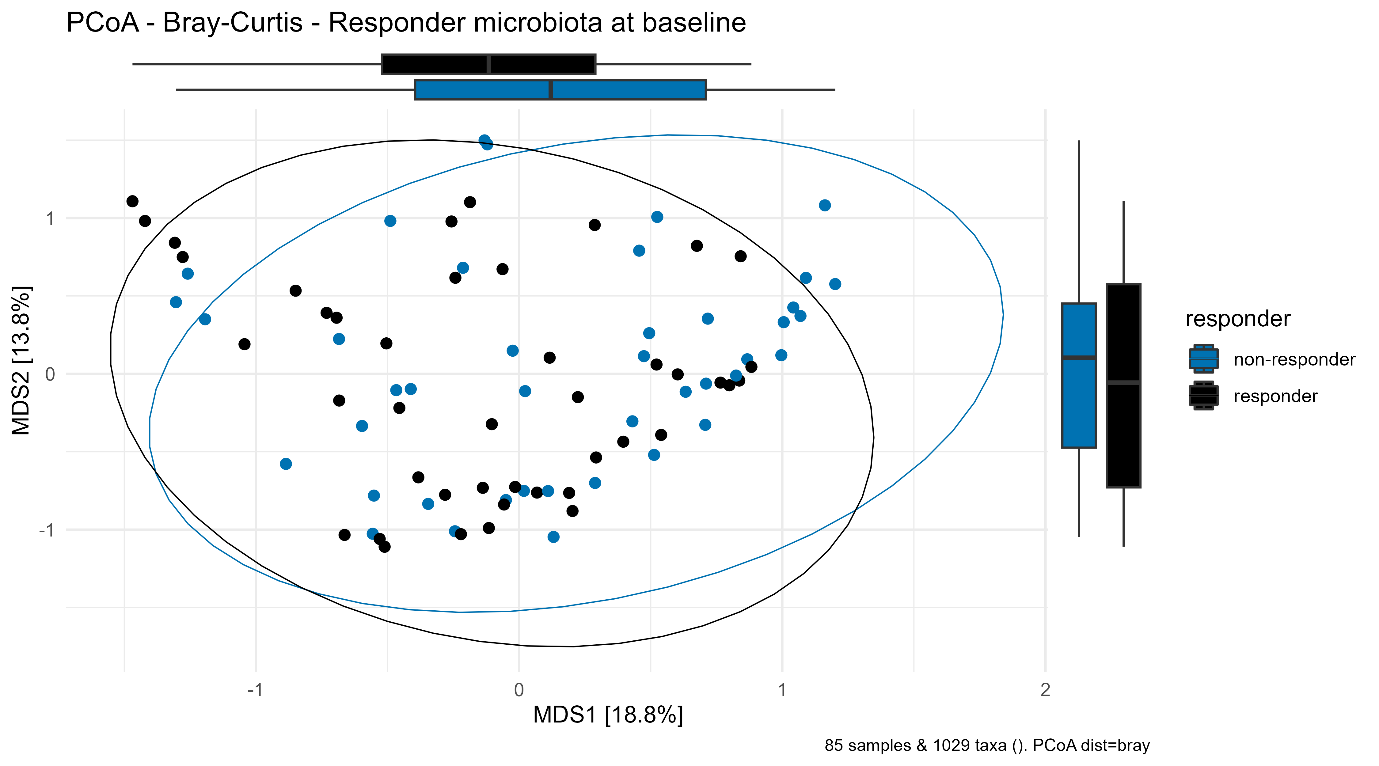
**

**Supplementary Figure 4.** Multi-dimensional scaling (MDS) plot of Bray-Curtis distances at baseline for responders (n=43) and non-responders (n=42) of the intervention, both intervention groups combined. Baseline microbiota composition was significantly different between responders and non-responders based on PERMANOVA analysis of Bray-Curtis distances (*p*=0.04)


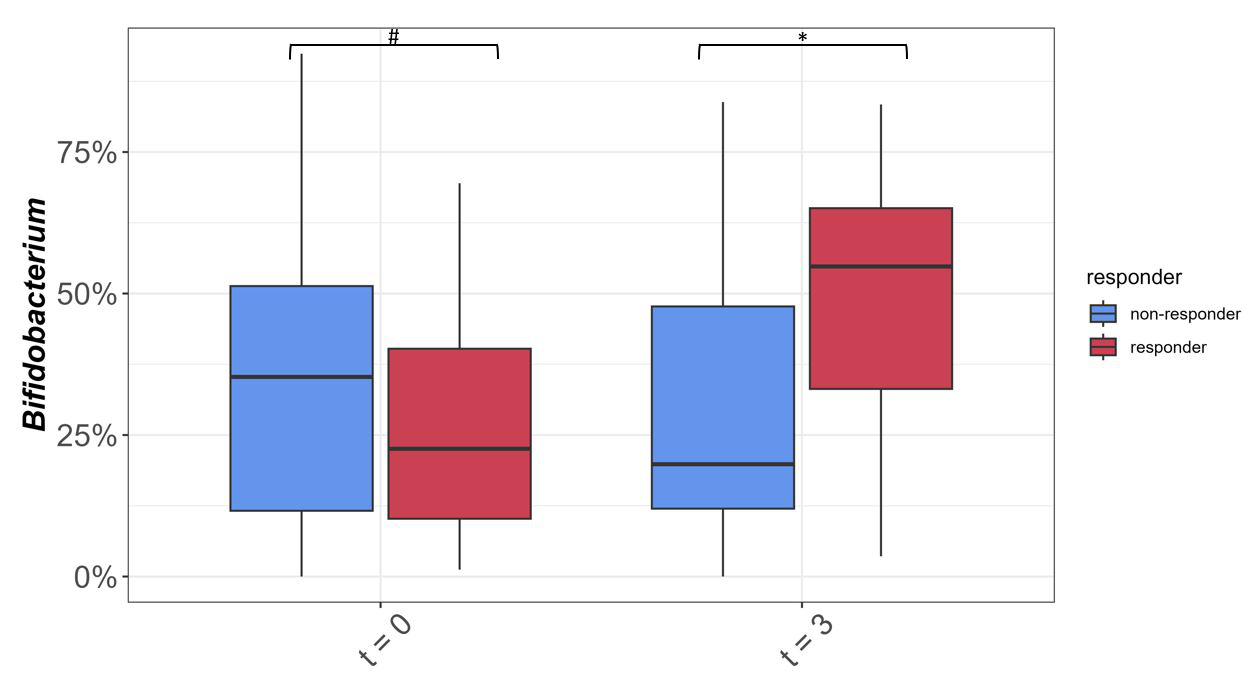


**Supplementary Figure 5.** Boxplots of relative abundance of *Bifidobacterium* (%) in fecal samples taken at baseline (t=-0) and at the end of the 3-week intervention period (t=3) for responders (n=43) and non-reponders (n=42), both intervention groups combined. #Baseline levels of *Bifidobacterium* tended to be lower in responders compared to non-responders (*p*=0.10). *The relative abundance of *Bifidobacterium* after intervention was significantly different between responders and non-responders (p<0.01).


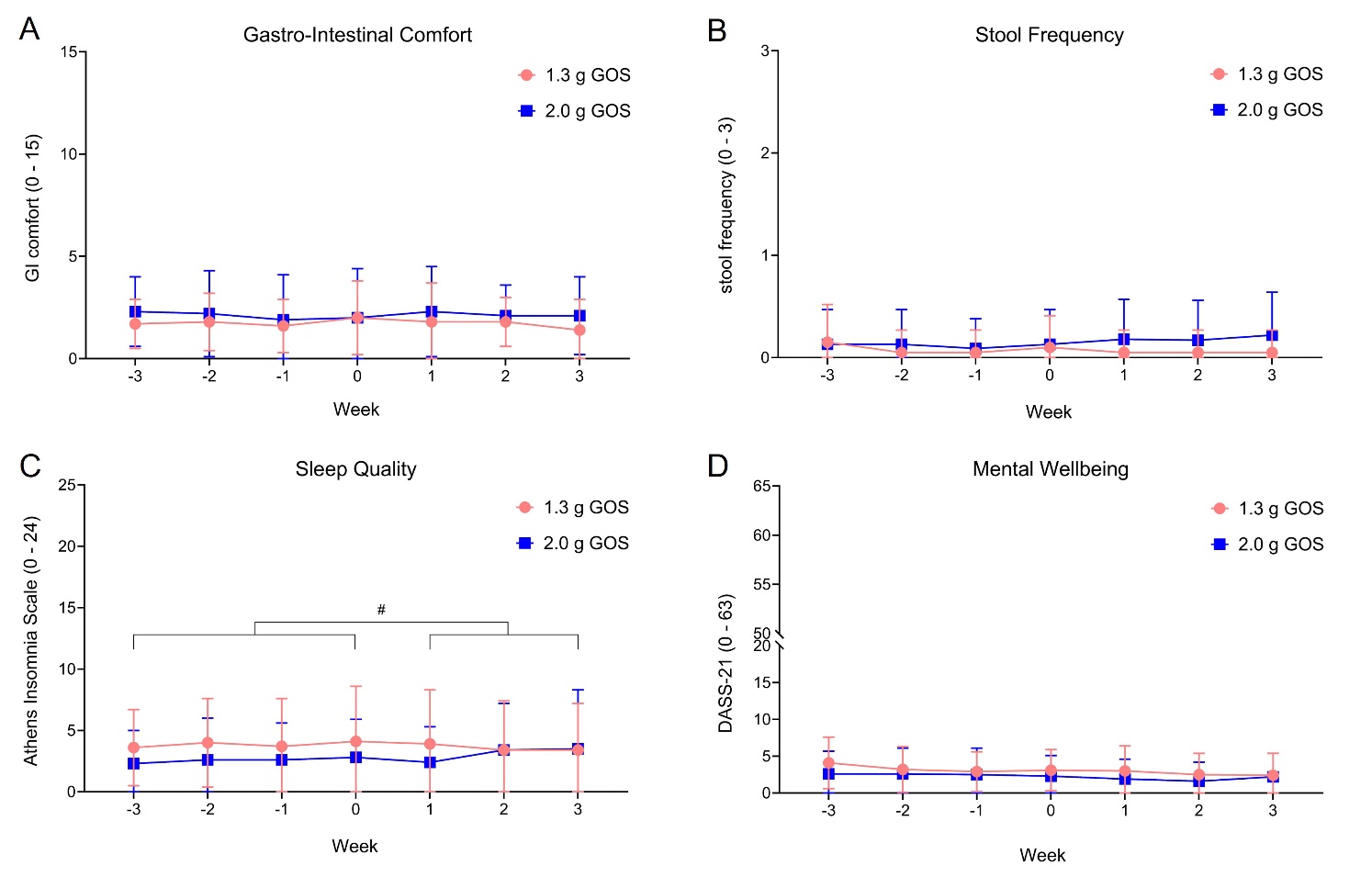


**Supplementary Figure 6.** Wellbeing parameters gastro-intestinal comfort (A), stool frequency (B), sleep quality (C) and mental wellbeing (D), within the responders of the 1.3 g GOS group (n=20) and the 2.0g GOS (n=23) group. Week -3 to week 0 is control period; week 1 to week 3 daily GOS supplement is used. Scoring ranged from 0 to 15 for GI comfort, from 0 to 24 for sleep quality and from 0 to 63 for mental wellbeing. Higher scores indicating more complaints. Stool frequency ranges from 0 (>5 times a week) to 3 (<1 time a week). # A trend (*p* = 0.07) was observed in sleep quality for 1.3g GOS only*.*

## Supplementary Tables

**Supplementary Table 1.** The number of subjects and their responder status in each of the clusters from the HCPC analysis.

| Cluster | Non-responder | Responder |
| --- | --- | --- |
| 1 | 17 | 3 |
| 2 | 10 | 27 |
| 3 | 5 | 9 |
| 4 | 10 | 4 |
